# Novel modulatory effects of neurosteroids and benzodiazepines on excitatory and inhibitory neurons excitability: a multi-electrode array recording study

**DOI:** 10.3389/fncir.2012.00094

**Published:** 2012-11-27

**Authors:** Giulia Puia, Francesca Gullo, Elena Dossi, Marzia Lecchi, Enzo Wanke

**Affiliations:** ^1^Department of Biomedical Sciences, University of Modena and Reggio EmiliaModena, Italy; ^2^Department of Biotechnology and Biosciences, University of Milano-BicoccaMilan, Italy

**Keywords:** neurosteroids, benzodiazepines, GABA_A_ receptor modulators, neocortical cultures, multi-electrode array

## Abstract

The balance between glutamate- and GABA-mediated neurotransmission in the brain is fundamental in the nervous system, but it is regulated by the “tonic” release of a variety of endogenous factors. One such important group of molecules are the neurosteroids (NSs) which, similarly to benzodiazepines (BDZs), enhance GABAergic neurotransmission. The purpose of our work was to investigate, at *in vivo* physiologically relevant concentrations, the effects of NSs and BDZs as GABA modulators on dissociated neocortical neuron networks grown in long-term culture. We used a multi-electrode array (MEA) recording technique and a novel analysis that was able to both identify the action potentials of engaged excitatory and inhibitory neurons and to detect drug-induced network up-states (burst). We found that the NSs tetrahydrodeoxycorticosterone (THDOC) and allopregnanolone (ALLO) applied at low nanomolar concentrations, produced different modulatory effects on the two neuronal clusters. Conversely, at high concentrations (1 μM), both NSs, decreased excitatory and inhibitory neuron cluster excitability; however, even several hours after wash-out, the excitability of inhibitory neurons continued to be depressed, leading to a network long-term depression (LTD). The BDZs clonazepam (CLZ) and midazolam (MDZ) also decreased the network excitability, but only MDZ caused LTD of inhibitory neuron cluster. To investigate the origin of the LTD after MDZ application, we tested finasteride (FIN), an inhibitor of endogenous NSs synthesis. FIN did not prevent the LTD induced by MDZ, but surprisingly induced it after application of CLZ. The significance and possible mechanisms underlying these LTD effects of NSs and BDZs are discussed. Taken together, our results not only demonstrate that *ex vivo* networks show a sensitivity to NSs and BDZs comparable to that expressed *in vivo*, but also provide a new global *in vitro* description that can help in understanding their activity in more complex systems.

## INTRODUCTION

Neurosteroids (NSs) such as tetrahydrodeoxycorticosterone (THDOC) and allopregnanolone (ALLO) are synthesized endogenously within the brain and potently modulate GABAergic synaptic transmission (Puia et al., 1990, 2003; Belelli and Lambert, 2005); however, their global function in regulating network firing activity is still largely unclear. The levels of NSs in the brain vary in different regions and so does the expression of the synthetic enzymes. The estimated average brain NS concentration is generally not higher than 10 nM, although it may change under different physiological and pathological conditions (Reddy and Rogawski, 2002; Weill-Engerer et al., 2002; Serra et al., 2000; Maguire and Mody, 2007). For example, mild (Purdy et al., 1991) and chronic (Matsumoto et al., 2007) stress increase the brain levels of NSs and it was recently demonstrated that new synthesis of these compounds is essential for the physiological response to stress (Sarkar et al., 2011). Conversely, a decrease in NS levels was detected in post-traumatic stress disorder, in major depression (Pinna, 2010), in Alzheimer’s and Parkinson’s diseases and in amyotrophic lateral sclerosis (Luchetti et al., 2011).

In the neocortex, the ALLO and THDOC synthesizing enzymes, respectively, 5α-reductase (5α-R) type I and 3α-hydroxysteroid dehydrogenase (3α-HSD), co-localize in glutamatergic but not in GABAergic neurons (Agis-Balboa et al., 2006). Therefore, excitatory neurons that co-express GABA_A_Rs and NS synthetic enzymes can be regulated in an autocrine manner (Gunn et al., 2011). However, it is not clear whether these cells are exposed to a stable extracellular concentration of NSs (Puia et al., 2003) that guarantees a tonic level of network inhibition, or the neuronal network is finely tuned by local stimulation of NS synthesis.

These notions prompted us to investigate the effects of nanomolar NS concentrations in networks formed by cells acutely dissociated from post-natal mouse neocortex. In these cultured networks, the spontaneous reverberating activity of excitatory and inhibitory neurons can be simultaneously recorded with multi-electrode arrays (MEAs) for days or even weeks (Gramowski et al., 2004; Selinger et al., 2004; Van Pelt et al., 2004; Arnold et al., 2005; Tateno et al., 2005; Eytan and Marom, 2006; Wagenaar et al., 2006; Gullo et al., 2009, 2010; Baltz et al., 2010). The MEA technique was considered to be an optimal choice for the present experiments, because it has also been demonstrated useful for recording and analyzing network activity from acute normal or epileptic brain slices (Mapelli and D’Angelo, 2007; Berretta et al., 2010; Gonzalez-Sulser et al., 2011), and from organotypic co-cultures (Dossi et al., 2012). In addition, since NSs have often been referred to as “endogenous benzodiazepines” we were interested to compare their effects with those of some established BDZ ligands, a class of widely prescribed drugs used as anxiolytics, hypnotics, sedatives, and anticonvulsants. The action of both NSs and BDZs is known to be mostly mediated by the potentiation of GABA neurotransmission, pre- and postsynaptically (Jo et al., 2011; Kim et al., 2011). We thus studied the effects of these drugs by analyzing the average response of excitatory and inhibitory neuronal clusters, measured as changes of excitability (spikes-per-engaged neuron) during the up-states (bursts), which are known to be statistically homogeneous in control conditions (Gullo et al., 2010).

As expected, both NSs and BDZs strongly decreased cell firing at low nanomolar concentrations. More interestingly, they also produced long-lasting changes in the network connectivity. The effects of THDOC and ALLO persisted for hours and could be considered a form of long-term depression (LTD). Clonazepam (CLZ) and midazolam (MDZ) also decreased network excitability, but only MDZ produced LTD. Application of NSs and BDZs altered the network activity by increasing the stationary heterogeneity among bursts. In particular, we observed the random appearance of novel up-states, characterized by excitability features and engaged neurons different from those observed in the absence of the drugs. Taken together, our results provide new light on the important regulatory role played by endogenously released allosteric NS modulators of GABA_A_Rs on local neocortical networks.

## MATERIALS AND METHODS

### CELL CULTURES

Primary cultures of cortical neurons were prepared as previously described (Gullo et al., 2009). Briefly, all of the cerebral cortices (excluding the hippocampus) were removed from decapitated post-natal mice (P1–P3), cut into 1 mm^3^ pieces, and digested by trypsin (0.15%) and DNAse (10 μg/ml) at 37^°^C for 20 min. After enzyme digestion, cells were mechanically dissociated by means of trituration, and plated at densities of 600–900 × 10^3^ cells/ml on glass coverslips (for immunocytochemistry) or MEA Petri dishes pre-coated with polyethyleneimine 0.1% (wt/vol) and laminin 20 μg/ml (30 μm diameter ITO electrodes spaced 200 μm apart, Multichannel Systems, Germany). After 3 h incubation, the plating medium was replaced by neurobasal medium (NB) with B27 (Invitrogen, Italy), glutamine 1 Mm, and bFGF 10 ng/ml, and the culture was maintained at 37^°^C in 5% CO_2_. One-half of the medium volume was replaced every 3 days. The cultures in MEA dishes were covered with gas-permeable covers (MEA-MEM, Ala Scientific Instruments, Inc., USA) throughout the culture period.

### DRUG APPLICATION: GENERAL ASPECTS

As previously described (Gullo et al., 2009), we report results obtained within a few hours after the MEA dish positioning into the incubator, which can thus be considered at the steady-state. The recording area in our MEA dishes was ~2 mm^2^, and we assume that the average number of neurons (plus glia) was of the order of ~6000 cells; the average space between cells was therefore relatively large. The NS drugs ALLO and THDOC, the BDZs CLZ and MDZ, finasteride (FIN) and the GABA_A_R antagonist gabazine (GBZ, also known as SR95531), were all purchased from Tocris (UK) and kept as frozen stock solutions in distilled water (or DMSO <0.1%) at -20^°^C, until diluted as appropriate with MEA culture medium before each experiment. All experiments were performed by adding the drugs in volumes that were always <1% of the total conditioned media volume bathing the neurons. The dose–response curves were obtained by adding increasing drug concentrations every 10 min, leaving at least 2 min to allow the drug to diffuse throughout the culture dish. When indicated, a wash-out was carried out with a solution pre-conditioned by the same network under control conditions.

### RECORDINGS, WAVEFORM ACQUISITION, AND SORTING

Data were recorded as previously described (Gullo et al., 2009). Briefly, analog signals sampled at 40 kHz were recorded at 36^°^C in CO_2_-controlled incubators using MEA-1060BC or 1060INV pre-amplifiers (bandwidth 1–8000 Hz, Multichannel Systems) connected to a MEA Workstation (bandwidth 100–8000 Hz, Plexon Inc., USA). Data were sorted into timestamp files by the MEAWorkstation Sorter software (MEAWS, see details below) and cleaned of artifacts using the OFFLine Sorter program (Plexon Inc.). Unless otherwise specified, we used 12–22 days *in vitro* (DIV) MEA dishes showing no fewer than 25 active electrodes and no fewer than 60 units. This age interval was considered the best to ensure fairly stable activity: the average MEA spike waveform firing rate in the controls was 68 ± 9.2 Hz (*n* = 18), in line with the values reported by others (Wagenaar et al., 2006). The MEAWS capture acquisition procedure was carried out in a window of 1.2 ms, in accordance with a previously described mixed amplitude/duration criterion (Gullo et al., 2009, 2010). The electrodes responding irregularly during the experiments were excluded from the analysis. Subsequently, to avoid artifacts, threshold was readjusted and signals were cleaned of spikes whose inter-spike interval was shorter than the pre-fixed 2.5 ms refractory period, by the OFFLine Sorter program (Plexon Inc.).

Next, during the principal component analysis (PCA)-based waveform sorting and for multi-unit electrodes, we applied one of the following procedures: (i) spike removal with a Mahalanobis threshold in the range 1.8–1.4; we concurrently checked that the *P*-value of multivariate ANOVA sorting quality statistics was <0.01 amongst the identified units; (ii) when the previous procedure led to excessive spike invalidation, we manually removed the spikes invading the adjacent unit ellipsoids (the latter method was very effective in decreasing the *P*-values, with only a limited number of erased spikes).

### NEURONAL CLUSTER IDENTIFICATION

The method of neuronal classification is described in Gullo et al. (2009), 2010 and Becchetti et al. (2012). Briefly, we identified the bursts with the same procedure used in Neuroexplorer software for all the bursts in which more than two spikes were identified. For all the cases in which two spikes were observed, we assigned a burst duration (BD) equal to their ISI and spike number (SN) of 2; moreover, for the cases in which one spike was observed, we assigned a BD of 2 ms and a SN of 1. This decision was based on the following reasoning: (1) we always observed that units in which sometimes one spike was observed, are characterized by many other bursts in which the unit was eliciting two or more spikes: (2) these units had always average SN values higher than 2; (3) it is known that pyramidal neurons normally fire few spikes, because they are under the control of feedback and feed-forward inhibitory neuron loops; (4) this “unreliable” behavior was typical of CNS neurons and of repeated stimulations observed *in vivo* and thus should be considered physiological also *in vitro* when a network is reverberating; (5) if this type of analysis were not present in our procedures, we suffered a strong “underestimation” of average values of SN; (6) our networks were silent during the down states, i.e., the intervals between bursts, and we disregarded the units (1–2 in each network) that fired continuously; (7) all these points were confirmed by the novel type of analysis explained in Gullo et al. (2012).

For each identified unit and each burst, the following characteristics were computed in defined time segments: the autocorrelation function (ACF), the BD, the SN, the spike rate (SR), the intra-burst spike rate (IBSR), the inter-burst intervals (IBIs), and the Fano factor (FF; Teich, 1989; Baddeley et al., 1997). We classified the neurons on the basis of an unsupervised learning approach consisting of data reducing PCA based on FF as a feature (Becchetti et al., 2012), followed by the K-means clustering procedure (Duda et al., 2000). We did not use a clusterization based on the classical spike-width computation (Constantinidis and Goldman-Rakic, 2002) because it was impossible to find a bimodal distribution of the spike-width data (Becchetti et al., 2012). After the clusterization based on FF, it was possible to recognize a bimodal pattern in the FF, BD, SN, and IBSR histograms. This result demonstrates that crucial physiological properties were highly different in the clusterized neurons. The large differences in these burst metrics was the basis for adopting FF as the best feature to clusterize neurons. Cluster processing was enriched by means of an outlier removal procedure that discarded the units whose Mahalanobis distance from the centroid of the cluster was greater than a fixed threshold (we used 1.4). As previously described (Becchetti et al., 2012), these procedures normally identified two statistically different clusters composed of variable numbers of excitatory (~50–80) and inhibitory (~15–25) neurons whose ratio always fitted the ratio present in the neocortex (Sahara et al., 2012).

### ADVANCED BURST CLASSIFICATION INTO STATES

The global network burst structure was analyzed with standard techniques (see Ham et al., 2008) as well as procedures recently developed by us (Gullo et al., 2012). Briefly, we applied a running window of variable duration (5–100 ms) in order to search for the start of the up-state and collect the spikes. The new procedure consisted of performing a classification of network states controlled by a PCA based on the following features: SNTH (spike number time histogram), neuron number (NN), and BD. The statistical significance of the classification was assessed by means of a two-sample paired *t*-test (*P* < 0.05). The states with a percentage occupancy (PO) of <4% in the time segment were discarded. To illustrate how a given network can change its mode of generating bursts, a raster plot of a typical experiment is shown in **Figure 2**. Two states were identified in the bottom window (100 s), which illustrates the results obtained in the presence of NS. The upper window refers to the control condition (without NS). After identifying the statistically different states (in the time segment of interest, TSOI), we plotted several histogram types (associated with the two clusters of neurons): (1) the probability density function of finding 1, 2, 3, *i*-th spikes (FSH) and its cumulative probability (cFSH), in order to investigate the neuronal firing mode; (2) the SNTH, the NNTH (the time-histograms of the number of engaged neurons, for each time bin) and the ratio of these histograms, here called “excitability” time histogram (EXTH; Gullo et al., 2012).

To help the reader compare the different results of our dose–response tests, we plotted, for each time-segment, the cFSH data of both control (thin lines) and treatment (thick lines). For the EXTHs, we plotted the ratio EXTH [at *i*-th drug value]/EXTH[control], which estimates the fractional effect and thus allows us to average data from different experiments. In **Figures 6 and 8** we used a simplified method consisting of averaging separately the data histograms for each state. In this way, we obtained directly the excitability and number of engaged neurons, as plotted in the figures. For each state, data were weighted by their PO, in order to estimate directly their relative contribution to each time segment.

### DATA ANALYSIS AND STATISTICAL ANALYSIS

The data were analyzed and the figures prepared using OriginPro 7.0 software (OriginLab Co., Northampton, MA, USA). All of the data are expressed as mean values ± SEM, with *n* indicating the number of experiments. Statistical significance was assessed using a paired Student’s *t*-test at the indicated significance level (*P*). If the data normality test was not satisfied, the Kolmogorov–Smirnov test was used.

## RESULTS

**FIGURE 1:**
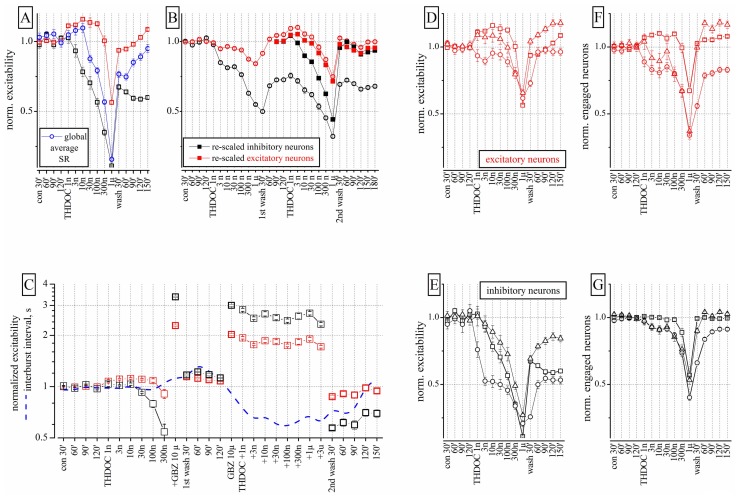
**Effects of THDOC on network excitability: reproducibility, action of GBZ and the plot of engaged neurons**. **(A)** Plot of the normalized excitability of excitatory (red) and inhibitory (black) neuronal clusters in control (cont 30, 60, and 90 min), after cumulative application of THDOC (1 nM to 1 μM) and after wash-out (wash 30, 60, 90, 120, and 150 min). The normalized averaged spike rate (SR: blue line) is shown in the diverse conditions and after >2 h of wash-out; nominal mean SR for the 68 excitatory (and 13 inhibitory) exemplary neuron clusters were 0.15 ± 0.02 (1.2 ± 0.1) Hz and a total global SR of ~25 Hz (*n* = 81). In control, the average excitability (over 30 min) used for data normalization was for inhibitory clusters 18.9 ± 0.13 spikes/neuron (*n* = 23 cells, 2 outliers) and for excitatory clusters 3.4 ± 0.03 spikes/neuron (*n* = 75 cells, 11 outliers). Average inter-burst interval (IBI) was 24.2 ± 3 s. Each concentration point is the averaged excitability recorded in a 600-s time segment. **(B)** Data from an experiment in which THDOC was applied in sequence two times after a 120-min wash and after the second wash-out of 3 h. Closed symbol data are the open symbol data but re-scaled to 1 under the assumption that the first wash-out was the control of the second application of THDOC (notice that the data of the second wash-out are aligned with those of the first wash-out). These results suggest that THDOC effects are reproducible except for the long-term decrease of inhibitory neuron excitability (by ~0.5) observed during the four wash-out segments (wash 30, 60, 90, and 120 min). Notice that recovery did not show any type of LTD effect. **(C)** The THDOC response was blocked by GBZ 1 μM. Excitability plot (red: excitatory; black: inhibitory neurons) and inter-burst interval plot (IBI, blue dashed-line) during control, THDOC (1–300 nM), GBZ 10 μM and again THDOC (1–300 nM), then wash-out for 30–150 min. After wash-out, the excitability of inhibitory neurons was depressed while that of excitatory neurons recovered to control values. **(D,E)** Plots of normalized excitability for inhibitory and excitatory clusters in three different experiments. **(F,G)** Plots of engaged neurons in the same experiments. Different symbols represent data derived from different neocortical cultures (*n* = 3). The ratio of excitatory and inhibitory neurons was 67/13, 84/14, and 86/16 respectively in the three experiments.

**FIGURE 2:**
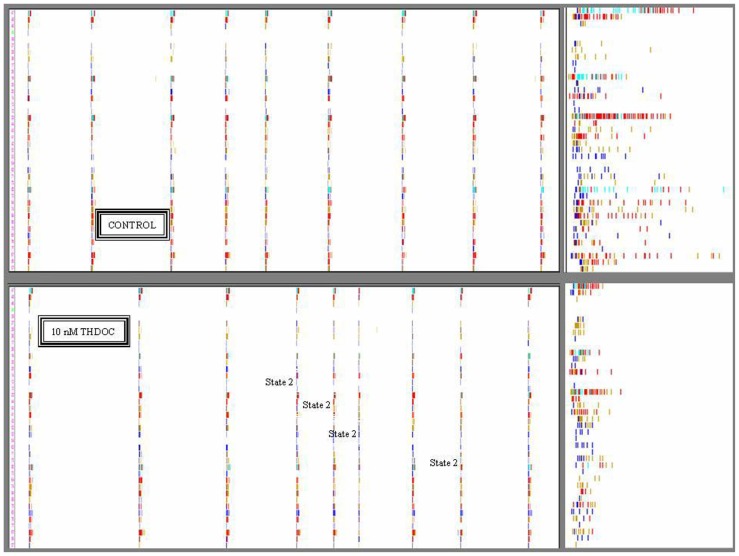
**Timestamps raster plots of spontaneously reverberating network bursts in control and during 10 nM THDOC**. The nine columns in the upper and lower panels indicate the bursts. The small vertical bars in the rows correspond to activity-timestamps from each electrode and different colors could be seen when more than one unit was identified. Time window length was 100 s. *Upper*: Data from a control time-segment in which up-states had a maximal duration of ~1 s. The right inset illustrates the details of firing in one burst in control on a 1-s time window scale. No short bursts were seen during several hours of recording. *Lower*: Data recorded in the time-segment after application of 10 nM THDOC. Notice that the legends indicate the bursts assigned to state 2 (short duration ~0.5 s; 4th, 5th, 6th, and 8th) by the software-based analysis. In the right inset (time window 1 s), it is particularly clear that the duration of the sixth burst was much shorter than those not assigned to state 2. Notice also in the insets that some neurons had always either a short or a long series of spikes corresponding to excitatory or inhibitory cells, respectively. Data correspond to the experiment analyzed in Figure 4 lower at 10 nM THDOC. Image windows obtained from the OffLine Sorter software (see Materials and Methods).

The firing activity of spontaneously reverberating networks of cortical neurons in control, after drug administration and during wash-out were analyzed as described in Section “Materials and Methods.” The results of a representative dose–response experiment with THDOC (1 nM to 1 μM) are shown in **Figure 1A**, which plots the normalized average spikes-per-engaged neuron in a burst. Moreover, a representative picture of the network reverberation bursting is given in **Figure 2** in the form of neuronal raster plots. In **Figure 2**, the upper and lower panels show, respectively, nine bursts recorded during 100 s in control and in the presence of 10 nM THDOC. Notice that in the right insets the time axis is 1 s long. The statistical properties of the bursts will be fully described later (see **Figure 4**).

### LOW CONCENTRATIONS OF THDOC EXERT DIFFERENT EFFECTS ON IDENTIFIED INHIBITORY AND EXCITATORY NEURON CLUSTERS

Low concentrations (1–30 nM) of THDOC applied cumulatively, progressively decreased the excitability of identified inhibitory neuron clusters, whereas the activity of identified excitatory cells was relatively unchanged or in some cases slightly increased. Higher concentrations of THDOC however, affected excitability in both inhibitory and excitatory clusters and the network was silenced at concentrations greater than 1 μM. Interestingly, several hours after wash-out, a persistent depression of inhibitory neuron firing was observed, which resembled LTD (**Figure 1A**). The average global SR (normalized to control) of all neurons (blue line) was also decreased by THDOC and only partially recovered after wash-out.

To study the properties of the LTD effect, we proceeded as follows. The sequence of THDOC doses was applied twice separated by a 120 min wash and followed by a final wash-out (*n* = 4). As illustrated in **Figure 1B**, in these experiments, only the excitatory neurons fully recovered from treatment. The effects of the second THDOC application partially mimicked those of the first one. To compare the effects of the two applications, we re-scaled and plotted the results obtained on the second application (closed symbols). Surprisingly, in this case, THDOC did not cause any further LTD. Taken together, these data suggest that the THDOC effects are characterized by a remarkable LTD of inhibitory neurons to a steady level, such that further applications show a complete reversibility (see closed symbols compared with the open symbols referring to the first THDOC modulation).

Next we investigated if GBZ (10 μM), a competitive antagonist of GABA_A_ receptors, could mask the NS effects of THDOC on our networks. As expected, GBZ applied in the presence of 300 nM THDOC completely reversed the NS effect (**Figure 1C**). Moreover, when the neuronal network was preconditioned with GBZ, a dramatic increase in excitability of both inhibitory and excitatory neuron clusters was observed. In this case, co-application of THDOC (1 nM to 3 μM) completely abolished the GBZ effect (see right part of **Figure 1C**). Nevertheless, after 2 h of wash-out from THDOC, a remarkable decrease (LTD) in the excitability of inhibitory neurons was again observed. Although THDOC *per se* did not alter the IBI (blue dashed line), the presence of GBZ during THDOC approximately halved the IBI in a reversible way, in agreement with the notion that the network activity was disinhibited.

These results however, had a degree of variability, as illustrated in **Figures 1D,E**, where excitability data from three different cultures are shown for both excitatory and inhibitory neurons. Although THDOC decreased excitability in all experiments, the effective concentration varied within the low concentration range (i.e., in 9 out of 13 experiments, THDOC was effective at 1 nM). To understand whether the reduction in excitability produced by THDOC was caused by a diminished firing activity of neurons (in each cluster) or by a decrease in number of neurons engaged in burst activity, we correlated the excitability (**Figures 1D,E**) with the number of neurons engaged in firing (**Figures 1F,G**). Comparing panels E and G of **Figure 1**, it is clear that at low THDOC concentrations, the responses of inhibitory neurons were highly variable, but the number of engaged neurons remained stable. At higher concentrations (300 nM and 1 μM THDOC) the values of excitability were more homogeneous and the number of engaged neurons decreased. On the contrary, at all THDOC concentrations, the activity of excitatory neurons correlated well with the number of firing neurons (**Figures 1D–F**). As noted above, inhibitory neurons were unable to completely recover their excitability for at least 3–4 h after wash-out.

### THE HETEROGENEITY OF BURSTS STUDIED BY STATISTICAL DESCRIPTION OF THE “STATES”

The persistent decrease in the excitability of inhibitory neurons in the presence of THDOC can be explained by the well-known potentiation of GABA inhibitory synapses produced by THDOC action (Belelli and Lambert, 2005). Since bursts are the brief times during which the network synapses are simultaneously active, we would expect that during this reverberating mode (i.e., resembling “matching” replications) the synaptic properties observed in control or in the presence of THDOC, should be modified accordingly in all of the “replications.” However, this was not the case, as THDOC produced a concentration-dependent heterogeneity among bursts (namely, the concept of “reverberation” of bursts was no longer valid).

**FIGURE 3:**
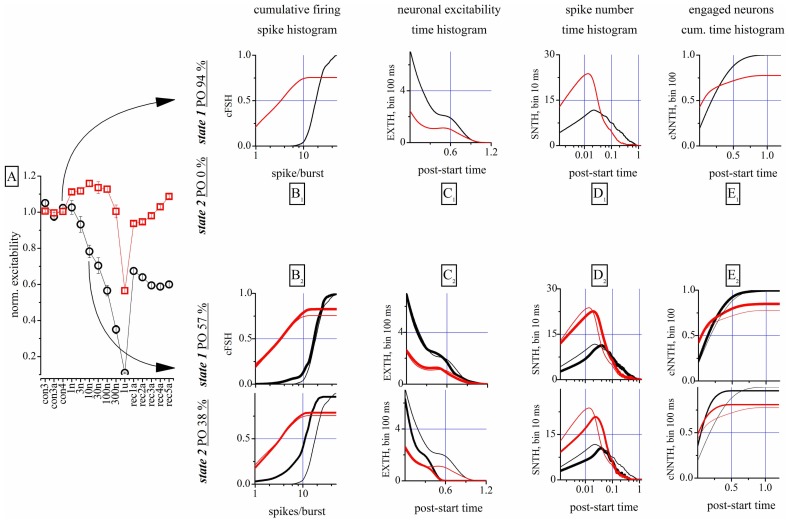
**Properties of the analysis of the network states**. **(A)** Global dose–response effects of THDOC obtained without the use of the “state” analysis. Arrows indicate the two time segments that were analyzed by using the “state” analysis. Only one state was identified in control, but in the 10 nM segment two states were identified. Red and black symbols show data derived from identified excitatory and inhibitory neuron clusters. (**B_1_,C_1_,D_1_,E_1_**) Plot of cFSH, EXTH, SNTH and NNTH histograms, respectively, during the control segment. The cumulative firing spike histogram (cFSH) plots describe how the number of spikes/burst are distributed in the excitatory or inhibitory clusters of neurons (left). The excitability time histogram (EXTH) provides a characterization in the time-domain (bins of 100 ms), about the average number of spikes elicited by engaged neurons. The average number of spikes time-histogram (SNTH) shows activity originating in each cluster at a resolution of 10 ms. The cumulative time histogram shows the average number of engaged neurons (cNNTH) in bursts (bin of 100 ms; (see Materials and Methods). Thus, different types of information on network activity could be studied: the first about the type of firing (average number of spikes in the burst) and the latter monitor the evolution during the burst life-time of the spikes elicited by each neuron (Gullo et al., 2012). (**B_2_,C_2_,D_2_,E_2_**) Plot of cFSH, EXTH, SNTH and NNTH histograms, respectively, during the 10 nM THDOC application. Same as in upper panels, but are shown also the plots of the second state (lowest part as indicated by the vertical left legend). Thin superimposed lines show control data from upper panels for comparison.

This is shown qualitatively in **Figure 2**, where 100 s-raster plots from 40 electrodes illustrate a typical firing pattern in control (upper, almost identical bursts) and in 10 nM THDOC (lower, not identical; see legend). To understand why the burst homogeneity observed in the controls was lost in the presence of THDOC, we applied a novel type of burst analysis that makes it possible to safely assign each network burst to one of two classes called “states,” which represent two statistically different modes of network connectivity (see Materials and Methods, and Gullo et al., 2012). This method captures the network activity features better than those that average the intrinsic heterogeneity of bursts. This is shown in **Figure 3**, in which the data obtained from two time segments (“control” and “10 nM THDOC”) are shown for a typical dose–response experiment. The global dose–response curves (**Figure 3A**) indicate that the two sets of data (arrows) were clearly distinguishable. Moreover, our “state” analysis (data in **Figures 3B–E**) shows that the statistical features we chose for state identification resulted in highly different burst modes recorded during THDOC application (in which two states were identified). Four types of histograms characterizing different statistics are shown for “control” and “10 nM” THDOC in the upper (labeled by subscript “1”) and lower (subscript “2”) panels of **Figure 3** right, respectively. In B_1_, C_1_, D_1_, E_1_ we plotted the cFSH, EXTH, SNTH, and cNNTH data for the controls (upward arrow) and the experiments with 10 nM THDOC (downward arrow). In control conditions, we only found one state (upper) with occupancy probability of 94 %, and the four histograms indicate that the curves corresponding to the two neuronal clusters were highly different. The control cFSH plot supports the notion that excitatory neurons are inclined to fire fewer spikes (range 1–10) compared to the inhibitory ones (range 10–30). Furthermore, because both cumulative plots reached a value of about 1, we conclude that almost all of the cells were engaged in burst activity (the same concept is illustrated in the time domain in panels E). The other histograms (EXTH and SNTH), evaluated in the time-domain in C and D, illustrate the time evolution of the two clusters. Application of 10 nM THDOC resulted in the appearance of two states (*t*-test, *P* < 0.05) with the following differences: (i) state 1 with properties very similar to those already observed in control, but with a PO that declined from 94 to 57% and (ii) a new state with PO 38% (state 2), characterized by significant changes with respect to state 1. This can be appreciated by comparing the thick lines (treatment) with the control data (thin lines), in the four histograms. In state 2, all bursts were much shorter (C_2_) and spike activity (D_2_) was delayed. No significant difference was instead observed in SR or IBI data, which turned out to be 1.14 ± 0.02 Hz and 16.3 ± 1.2 s in the controls, and 1.00 ± 0.09 Hz and 12.6 ± 0.7 s in 10 nM THDOC, respectively.

In the following paragraphs, we will mostly describe our results by using the global analysis, and give the results of the “state” analysis only when this adds significant interpretive value.

### MECHANISMS OF THDOC ACTION

**FIGURE 4:**
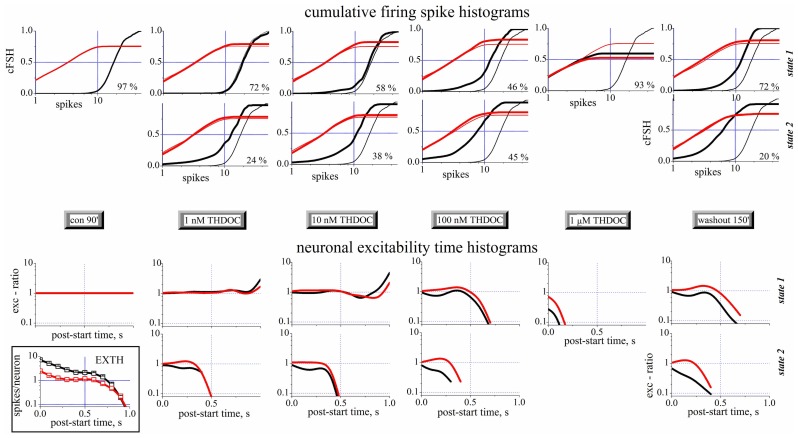
**Spike- and exc-ratio time-histograms during THDOC reveal the appearance of new states in the network**. Data from different time segments (see the gray legends in the middle). Data from excitatory and inhibitory neuron clusters are in black and red, respectively. For each time segment there are one or two plots depending if one (state 1, upper) or two (state 2, lower) states have been identified (see vertical legends at the right border). The percentage of the occupation (PO) of the two states is shown in each plot to the right. *Upper panels*: Cumulative spike histograms (cFSH). To compare each result (thick lines) obtained in the different time segments, the cFSH data found in control are superimposed as thin lines. *Lower panels*: Exc-ratio histograms (which report the ratio of EXTHs evaluated in the *i*-th segment with respect to control segment). In the inset below, for control, the excitability time histograms (EXTH) are shown. Control and wash-out segments had durations of 30 min (~110 bursts) while all the others had durations of 10 min (~40 burst). In control, only one state was detected, while after THDOC application, a second state appeared co-existing with the first one. Note that after 3.5 h of wash-out, the second state was still present. Data from one of the experiments shown in **Figures 1E–H**.

A complete analysis of the states in control, during the application of increasing concentrations of THDOC and after wash-out is shown in **Figure 4**. The upper part of this figure shows the cFSH corresponding to the different conditions. Only one state was detected in control condition. THDOC induced a second state, whose PO increased from 24 (1 nM THDOC) to 45% (100 nM THDOC), while the PO of state 1 decreased from 72 to 46%, at the same concentrations. At 1 μM THDOC, the network settled to a more homogeneous firing mode, which was dramatically different from the control and did not recover even after a long wash-out. The EXTH data are shown in the lower part of **Figure 4**. Each graph plots on a log-scale the ratio between the EXTH calculated at a given concentration and the EXTH in control condition, thus describing the on-line time-dependent performance of neurons. These graphs show that state 2 is merely characterized by a halving of the BD and only occurred in 24% of the bursts, at 1 nM THDOC. On the contrary, as illustrated by the cFSH results, at 1 μM THDOC all of the bursts were strongly shortened and wash-out was ineffective. Our quantitative description suggests that THDOC, by enhancing GABA_A_Rs currents, decreased the propensity of inhibitory neurons to fire. Interestingly, this effect only occurred in a fraction of the up-states, which depended on NS concentration. At 10 nM THDOC, the network could switch between two co-existing firing modes (state 1 and state 2), as was also highlighted in the raster-plot recordings shown in **Figure 2**. By increasing the THDOC concentrations from 1 to 100 nM, the state typical of the control (state 1) progressively disappeared and was substituted by state 2, which persisted after wash-out. Higher doses (1 μM) virtually silenced the network activity.

Taken together, this new type of description sheds light on the mechanisms underlying network activity. In this light, the LTD induced by NSs could be explained as: (1) a change of firing mode of inhibitory neurons in ~72% of the bursts (left-shift in cFSH) and (2) a qualitatively similar, but much stronger, decrease of activity in ~20% of the bursts. By contrast, small effects were seen in excitatory cells, whose bursts were however significantly shortened by NS treatment.

### IS THERE A SELECTIVE EFFECT OF THDOC ON “TONIC” GABA INHIBITION?

Previous studies performed in hippocampal and cerebellar granule cells suggested that THDOC behaves as a selective modulator of endogenous “tonic” inhibition mediated by δ subunit-containing GABA_A_Rs (Stell et al., 2003). It has been suggested that GBZ at concentrations of 100 nM selectively blocks phasic GABA currents (Stell and Mody, 2002). Therefore, we studied the effect on our networks of low concentrations of THDOC, in the presence of 100 nM GBZ.

**FIGURE 5:**
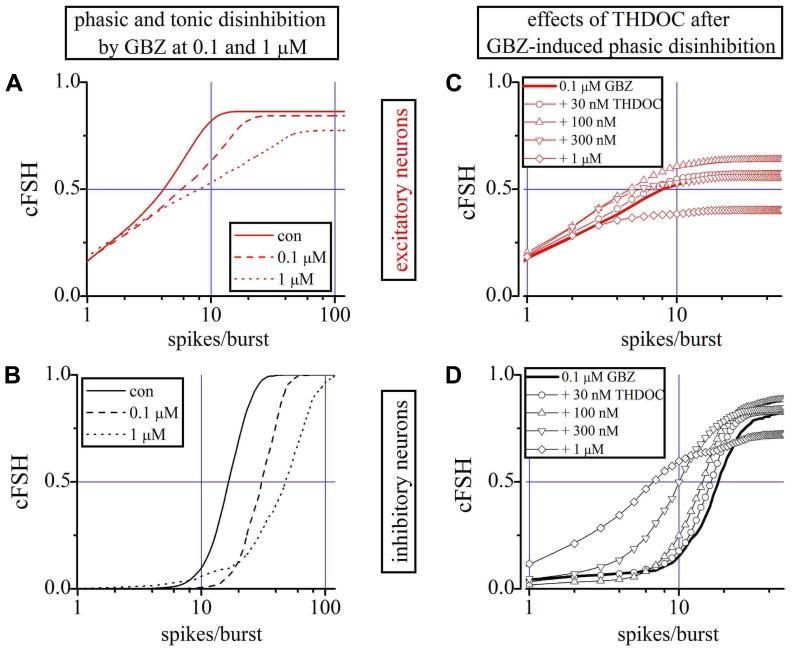
**Role of phasic and tonic network inhibition and the THDOC action on partially and totally disinhibited networks**. **(A,B)** Plots of cFSHs of excitatory and inhibitory neuron clusters (control, continuous line; 100 nM GBZ, dashed-line; 1 μM GBZ, dotted-line). An exemplary experiment in which 84 and 24 excitatory and inhibitory cells were identified. **(C,D)** Plots of cFSH of excitatory (red) and inhibitory clusters (black) after 0.1 μM GBZ (continuous line) and during THDOC application. Data obtained when THDOC was applied on top are shown with lines + symbols: open circles, upward triangles, downward triangles and diamonds indicate 0.03, 0.1, 0.3, and 1 μM THDOC on top, respectively. Notice in **(D)** that, with respect to the 0.1 μM GBZ pre-conditioning (continuous line) data are gradually left-shifted by the increasing THDOC concentrations, thus suggesting a corresponding decreased excitability and a decreased number of engaged neurons (line + diamonds) only at 1 μM THDOC (THDOC concentrations lower than 30 nM were out of action).

We first studied the effects of GBZ 100 nM and 1 μM alone on excitatory (**Figure 5A**) and inhibitory neurons (**Figure 5B**) by using the cFSH description. GBZ (100 nM) increased the network activity, suggestive of a substantial endogenous GABAergic “tone” in our cell cultures. The effect was more pronounced in inhibitory neurons, whose average SN per burst increased by 52% (*n* = 24). The mean increase was approximately 30% for excitatory neurons (*n* = 84). Application of GBZ 1 μM, to block both phasic and tonic GABAergic currents, increased the excitability of inhibitory neurons by 155% and that of excitatory cells by 85%. Furthermore, since IBIs were also shorter at high GBZ concentrations (see also **Figure 1C**), the SR of inhibitory neurons increased from 0.6 ± 0.05 to 0.9 ± 0.06 Hz with 100 nM GBZ and to 2.2 ± 0.11 Hz with 1 μM GBZ.

To analyze the effects of low concentrations of THDOC when only the phasic inhibition was blocked, we preconditioned a network with 100 nM GBZ (line) and then applied increasing concentrations of NS (**Figures 5C,D**). No significant effects were detected at concentrations of THDOC around 30 nM (circles), whereas the cFSH curve was increasingly shifted to the left by applying 100 nM (upward triangles), 300 nM (downward triangles), and 1 μM (diamonds). Therefore, when the phasic GABA response was blocked, the sensitivity of the network to THDOC decreased by approximately one order of magnitude compared to the control conditions.

### ALLO DECREASES EXCITABILITY IN BOTH NEURONAL CLUSTERS

**FIGURE 6:**
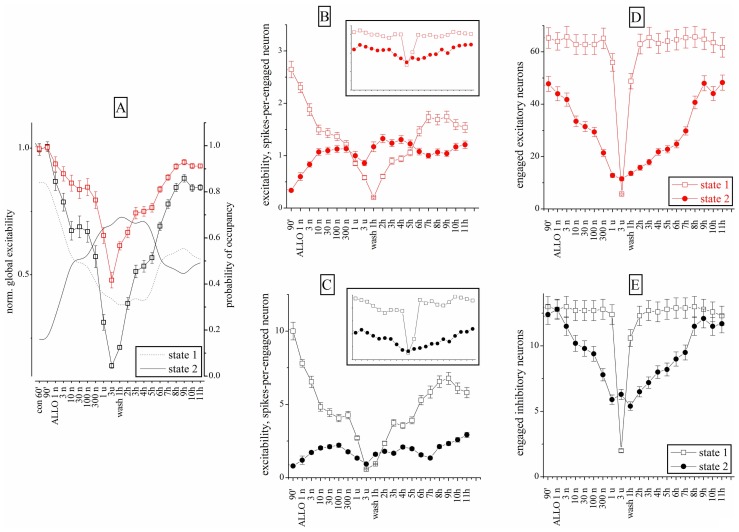
**Excitability and engaged neurons from “global” and “state” data analysis during ALLO dose–response experiments compared to control**. **(A)** Normalized global excitability dose–response curves for clusters of excitatory (red) and inhibitory (black) neurons during a long experiment. Superimposed lines show PO values for state 1 and 2 as indicated (right *y*-axis). **(B,D)** Excitability and number of engaged neurons for the excitatory cluster in the state 1 (open square) and 2 (closed circles). Inset: plots of the same data (same scales) but without taking into account the respective PO of each state. Notice that state 2 values are ~2/3 of state 1 values in the excitatory cluster. **(C,E)** Excitability and number of engaged neurons for the inhibitory cluster in the state 1 (open square) and 2 (closed circles). Notice that state 2 values are ~½ of the state 1 values in inhibitory cluster. Data segments from controls and wash-out had durations and number of bursts (in parenthesis) of 30 min (~120) and the ALLO segments had durations of 10 min (~40). At 3 μM ALLO only seven bursts were observed.

Similar experiments were carried out with ALLO (1–300 nM). **Figure 6A** shows the excitability changes observed after cumulative ALLO application in an 18 DIV neocortical culture. At low concentrations, ALLO reduced the excitability of inhibitory cells and, to a lesser degree, that of excitatory neurons. The global excitability was also followed during the recovery phase, for up to 11 h (**Figure 6A**) and the relative PO of state 2 is also given (the PO of state 1 is complementary). The states’ analysis is shown in **Figures 6B–E** in a simplified form (obtained by averaging the cFSH and EXTH data). We plotted, for each state and for each neuronal cluster, the excitability (**Figures 6B,C**) and the number of engaged neurons (**Figures 6D,E**; see Materials and Methods). The **Figures 6B,C** plots present data weighted by the PO. On the contrary, insets show non-weighted data and thus illustrate different aspects of the firing modes (i.e., the BD). No normalization was applied, in order to display the raw firing data of excitatory and inhibitory neurons clusters characterized, respectively, by weak (~3) and high (~10) excitabilities (spike/burst) and large (~60) and small (~16) cell numbers (range 2–75).

This analysis revealed that the excitability of both clusters in states 1 was characterized by a biphasic response, with a plateau between 10 and 300 nM ALLO. Interestingly, as shown in **Figures 6D,E**, the number of engaged neurons (state 1) was constant up to 1 μM. In state 2, the engaged neurons had a similar biphasic response. However, somewhat unexpectedly, their excitability increased only from 1 to 10 nM and remained almost constant at higher concentrations, with a slow recovery after 9–11 h of wash-out. The state analysis shows that this was mainly caused by a delayed recovery of state 1 probability, accompanied by a noticeable increase of both excitability and engaged neurons in state 2. The heterogeneity analysis indicates that all the state 2 bursts were characterized by a much shorter duration, compared with state 1. This suggests that the neurons’ ability to maintain the temporal connectivity may be impaired during the up-state.

On the whole, these results suggest that ALLO, by enhancing the GABA_A_ currents on both excitatory and inhibitory neurons, produced similar effects on state 1 mode and completely different effects on the connectivity of state 2. Only in this latter mode, the engaged neurons decreased, but not their excitability.

### THE BDZS CLONAZEPAM AND MIDAZOLAM DECREASED EXCITABILITY OF BOTH EXCITATORY AND INHIBITORY CLUSTERS

**FIGURE 7:**
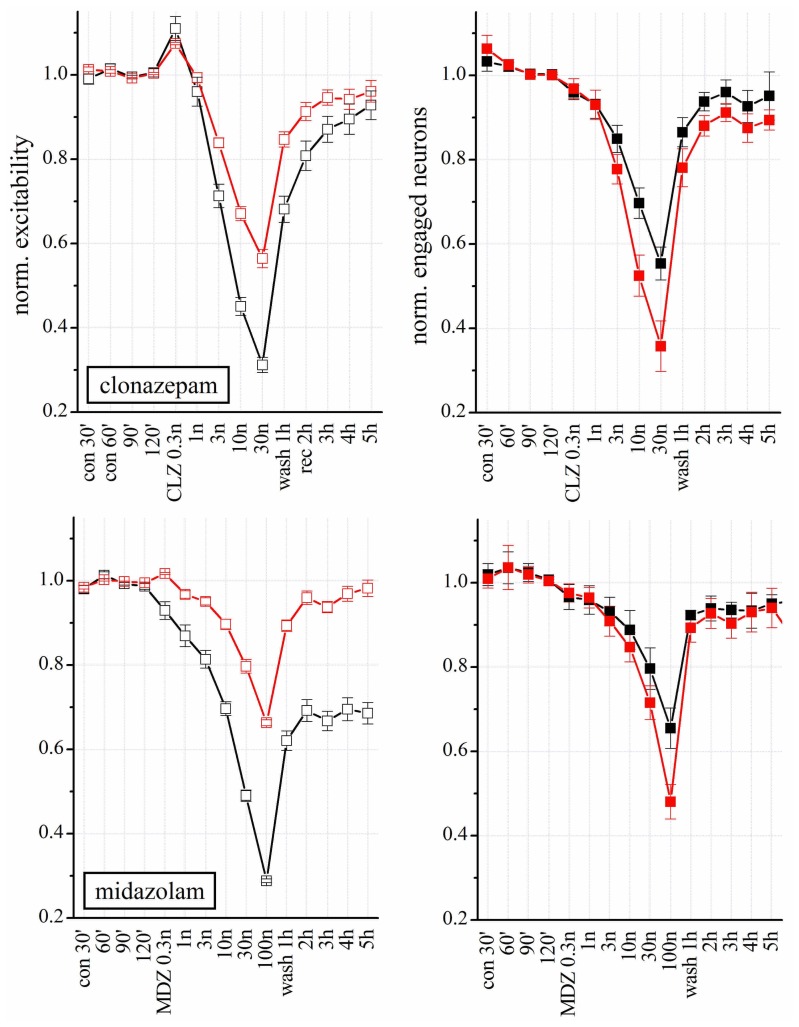
**Modulation of network activity by BDZs: differences in the effects of CLZ and MDZ**. *Upper*: Plots of the normalized excitability of excitatory (red) and inhibitory (black) neuron clusters in control (30, 60, 90, and 120 min), after increasing concentrations of CLZ (left) or MDZ (right) and after wash-out (wash 1–5 h). Notice that MDZ after 5 h wash-out still maintained an effect on inhibitory clusters while CLZ did not. Moreover, to completely silence the network activity, 100 nM MDZ was needed, while the same effect was reached with 30 nM CLZ. *Lower*: Plots of number of engaged neurons (normalized with respect to control). These data were derived from four complete double-experiments in which both BDZs were applied successively after a short 3 h-wash-out, and results were normalized and averaged for each plot (*n* = 8).

**FIGURE 8:**
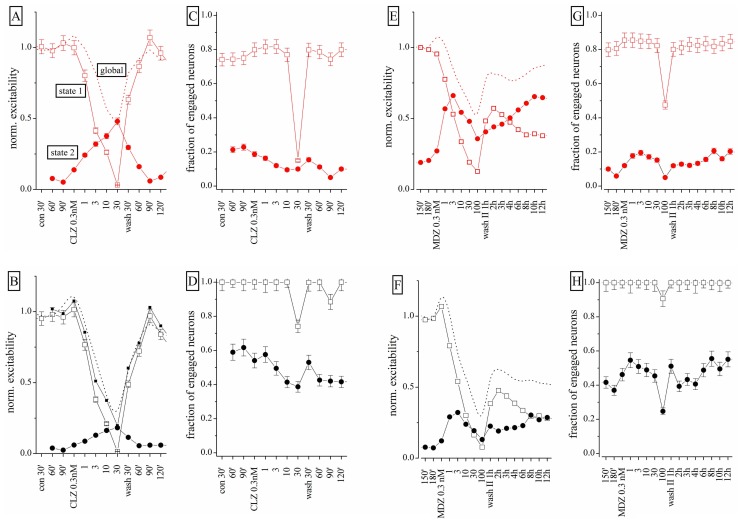
**Different effects of CLZ and MDZ at low nanomolar concentrations: a states analysis**. Data obtained from the same network on which increasing concentrations of CLZ **(A–D**) or MDZ **(E–H**) were applied after a 3-h wash-out; the wash-out II lasted 12 h and data displayed are for each hour. In the upper and lower panels, excitatory (red) and inhibitory (black) neuron cluster data are shown. Open square and closed circle symbols are related to state 1 and 2, respectively. **(A,B,E,F)** Plots are related to excitability and **(C,D,G,H)** plots are related to fraction of engaged neurons. Dotted lines are related to results obtained under the “global” analysis and not under the “state” mode analysis.

Clonazepam and MDZ also regulate GABA_A_Rs allosterically. Both affected network excitability in a concentration-dependent way (**Figure 7** upper panels; *n* = 8), but their potency was different. At 3 nM, CLZ decreased the excitability of inhibitory neurons by 30%, whereas the same concentration of MDZ produced a 20 % inhibition (but see also the experiments shown in **Figure 8**, carried out in the same network). At lower concentration (0.3 nM) CLZ produced a transient *hyper*excitability. Similarly to what was observed for NSs, the effects of both BDZs were much weaker on the excitatory clusters. Differently from CLZ, MDZ induced a persistent depression (LTD) of inhibitory cluster excitability, which lasted for hours after wash-out.

In **Figure 7**, we correlated the excitability changes caused by CLZ and MDZ (upper panels) with the number of engaged neurons (lower panels). Differently from what we observed with THDOC, the BDZ-dependent decrease in excitability was accompanied by a reduced number of engaged neurons, which recovered to the control condition after wash-out. On the other hand, the LTD induced by MDZ on inhibitory clusters was similar to that produced by THDOC and was not caused by a reduction of engaged neurons, but due to an intrinsic decrease in excitability.

To further study the action of BDZs, we performed experiments (*n* = 3) in the same dish at physiologically relevant concentrations, by applying CLZ before MDZ (*n* = 3), or *vice versa* (*n* = 4). When MDZ was applied first, we always observed the persistent LTD, insensitive to wash-out (not shown). We used the same simplified state analysis as illustrated in **Figure 6**, by plotting excitability and engaged neurons. Once again, the state 2 turned out to be seldom occupied in control condition, but frequently occupied in the presence of BDZ. State 2 was characterized by a BD that was less than half the one observed in state 1 bursts (corresponding to the control state). We compared dose–response curves of normalized excitability and fractional engaged neuron number for the two firing states in: (a) control; (b) in the presence of CLZ (**Figures 8A–D**); (c) after CLZ wash-out (3 h); (d) in the presence of MDZ (**Figures 8E–H**); (e) after prolonged MDZ wash-out (**Figure 8**).

Clonazepam concentrations between 0.3 and 10 nM produced significant occupancy of state 2, by decreasing the state 1 excitability in both neuronal clusters (**Figures 8A,B**). Interestingly, the excitability trend was opposite in the two state modes. The fractional number of engaged neurons (**Figures 6C,D**) did not change significantly, but at 30 nM CLZ the network was virtually silenced as the excitatory neuron percentage dramatically decreased from ~80 to ~15%, whereas the number of engaged inhibitory neurons scarcely changed (**Figure 8D**). The recovery was fast and almost complete, although state 2 did not completely disappear. After 3 h wash-out, MDZ was applied in a condition in which ~75 and ~30% of the bursts were sustained by state 1 and 2 respectively (not shown). MDZ produced visible effects on excitability only at concentrations higher than 1 nM (**Figures 8E,F**). At 100 nM MDZ was unable to fully silence the network. This BDZ maintained a fairly constant number of engaged neurons in each state (**Figures 8G,H**) and the trend of excitability changes (**Figures 8E,F**) was opposite for the two states. During the 12 h of wash-out, the excitatory neurons recovered 75% of their initial excitability, whereas inhibitory neurons’ excitability remained at around 30%, although the engaged neuron number was unchanged. The dashed lines shown in **Figures 6A,B** (for CLZ) and **Figures 6E,F** (for MDZ) display the global excitability data, independently from the state analysis. This summarizes our results in a way that, although masking the underlying network heterogeneity, captures the LTD induced by MDZ and the substantial recovery of the CLZ effect.

We conclude that the origin of the observed LTD in the network is most likely caused by irreversible changes, mainly occurring in the inhibitory synapses present on inhibitory neurons. Moreover, the observed excitability in the two firing states clearly suggests that these two modes identify two remarkably different sets of connectivity between neurons. Finally, by fitting the state 1 excitability data (both clusters) to a Hill-type curve, we found mean IC_50_ values for CLZ and MDZ of approximately 2.2 and 3.5 nM, respectively, which were not significantly different.

### DOES PRE-TREATMENT WITH FINASTERIDE ALTER THE EFFECTS OF CLZ AND MDZ?

**FIGURE 9:**
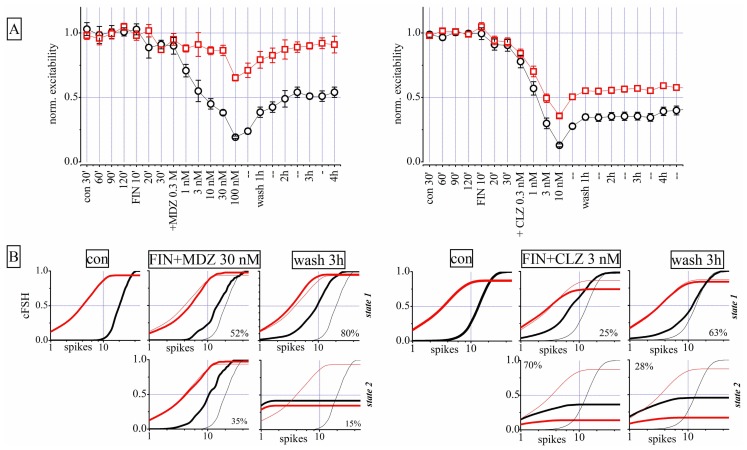
**Finasteride (FIN) pre-conditioning did not affect LTD produced by MDZ but induced an LTD effect after CLZ**. Left and right parts are related to two different exemplary experiments in which either MDZ or CLZ were applied after FIN, respectively. **(A)** Plots of normalized global excitability of excitatory (red) and inhibitory (black) neuron clusters in control (four samples of 30 min each), during the treatment with 1 μM FIN (three samples of 10 min each), after application of increasing concentrations of MDZ (left panel) and CLZ (right panel) and after 1, 2, 3, and 4 h of wash-out. Notice that the LTD effect on the inhibitory neuron cluster is still present after MDZ application and this is evident also after CLZ on both clusters. Only the excitability of the excitatory cluster of neurons recovered from MDZ, back to control values. **(B)** Plots of cFSH data of excitatory (red) and inhibitory (black) neuron clusters in control, after FIN + MDZ (left) or FIN + CLZ (right) and after wash-out. Notice that the superimposed thin lines indicate control data for comparison. Upper and lower panels are related to states 1 and 2, respectively.

As shown in **Figure 8**, both CLZ and MDZ decreased network excitability but only MDZ produced an LTD similar to that caused by THDOC. Tokuda et al. (2010) previously demonstrated that FIN, an inhibitor of NS synthesis, abolished the MDZ-dependent LTD in hippocampal slices. We thus tested whether FIN affected the long-lasting MDZ effect in our cortical preparations. We pre-treated neuronal cultures for 30 min with 1 μM FIN and then applied increasing concentrations of MDZ (*n* = 8). Surprisingly, under these conditions, MDZ still produced LTD (**Figure 9A**, left). In fact, pre-treatment with FIN increased the CLZ potency (*n* = 7), and after wash-out we observed LTD of both excitatory and inhibitory clusters. Application of FIN alone produced negligible effects.

The state analysis for FIN + MDZ shown in **Figure 9B** (left) supports the usual notion that the two firing states are present simultaneously and that, after wash-out, the inhibitory neurons were unable to regain their excitability (third column from left: cFSH plots of state 1 and 2). We obtained similar results by testing FIN + CLZ (**Figure 9B**, right), with the emergence of a highly-occupied second state ( fifth column) and an LTD of both neuronal clusters during wash-out (sixth column). In conclusion, we were unable to reproduce Tokuda et al.’s (2010) results on our system, with either MDZ or CLZ. The possible reasons for such a discrepancy are discussed below.

## DISCUSSION

We have demonstrated here that ~2 mm^2^ networks of neocortical neurons cultured for 12–19 days on MEAs are sensitive to nanomolar concentrations of NSs and BDZs. Therefore, we considered these cultures a realistic model to study the role of GABAergic inhibition at the integrative level. Networks comprising a few thousands of cells were monitored by continuously sampling the activity of ~100 neurons during drug application. In this way, we obtained distinct dose–response relationships for excitatory and inhibitory neurons, whose ratio in our cultures was considered close to the one observed in the cortex *in vivo* (Sahara et al., 2012). For the first time, physiological variables such as excitability and number of neurons engaged in bursts were described quantitatively by using robust statistical methods (Gullo et al., 2012). The quasi-homogenous activity of interconnected sets of principal cells and interneurons was described in terms of global activity, in control conditions. However, in the presence of drugs, the neuronal activity was split into heterogeneous modes (up-states) with occupancy probabilities strongly dependent on the drug concentration. These states had different elementary physiological properties (i.e., duration, excitability, engaged neurons, etc), that were fully characterized. Because no effects were seen on the action potential waveforms after drug application, our results suggest that changes in synaptic connectivity underlie the drug-induced processes we describe.

To the best of our knowledge, no studies are available on the modulation of network excitability by NSs and BDZs. In fact, identifying the neuron type and the up- and down-states simultaneously in hundreds of neurons, is not a trivial task. Other methods such as functional multi-neuron calcium imaging (Sasaki et al., 2007) have been applied to the study of CNS networks. However, MEA electrophysiology provides an exceptional temporal resolution that allows workers to properly sample both brief events (e.g., single spike waveforms) while recording very prolonged experiments (tens of hours) *in vitro* and *in vivo* (Truccolo et al., 2011). Moreover, with MEA recording, none of the typical problems offered by fluorophores, such as short-term toxicity and photobleaching, arise.

### EFFECTS OF THDOC AND ALLO ON NETWORK ACTIVITY

We first investigated the effects of an endogenous NS modulator, THDOC, on the global network activity. THDOC at physiological concentrations, selectively decreased inhibitory interneuron activity, whereas at concentrations higher than 100 nM it inhibited both excitatory and inhibitory clusters. The different sensitivity of these neuronal populations to NSs could depend on different factors, such as subunit composition and/or phosphorylation of the GABA_A_R, or local metabolism of the NS (Belelli and Lambert, 2005). Novel information was obtained from the continuous analysis of the network activity after long wash-out periods. Surprisingly, after several hours of wash-out (**Figures 1–4**) the excitability of inhibitory clusters did not recover.

The reasons for the long-lasting LTD-like effects of NSs are unknown. It is possible that in inhibitory neurons, THDOC activates post-translational modifications of the GABA_A_R that prolong the NS effect. For example, THDOC could regulate the protein kinase (PKCε or PKCγ) association with GABA_A_Rs, a process generally implicated in controlling receptor trafficking, as previously demonstrated for ethanol (Ron and Messing, 2013). Increasing evidence shows that regulation of a receptor recycling determines the efficacy of synaptic inhibition, further pointing to the necessity of deeper studies about the possible actions of NSs in synaptic membrane protein trafficking (Vithlani et al., 2011). Another possible explanation for the persistent effect of NSs is that inhibitory neurons could accumulate these compounds and then slowly release them during wash-out. In fact, NSs can readily diffuse into the cytosol and localize within the plasma membrane, thus locally regulating the GABA_A_R activity (Shu et al., 2004; Li et al., 2007; Akk et al., 2009). This idea accords with the fact that pre-treatment of our neuronal cultures with 10 μM GBZ prevented the effect of THDOC, but strongly decreased the activity of inhibitory neurons after 2 h of wash-out (see **Figure 1**). This suggests the following working hypothesis: in the presence of GBZ, GABA_A_Rs are blocked and the action of THDOC cannot be revealed. When GBZ is washed, the NS accumulated inside the cell can then be slowly released and thus modulate GABA_A_Rs.

To further clarify this point we designed experiments in which THDOC was applied twice (after a 2 h wash-out). The first THDOC application induced, as expected, a persistent depression of inhibitory neurons. After the second administration however, no further LTD was observed (see **Figure 1B**). This experiment prompted us to think that the first steroid application changed the response of inhibitory neurons in a stable manner, inducing a sort of “memory” in the network. We conclude that our experiments are perfectly reproducible if they do not show “memory” properties. On the contrary, when stable changes of the receptor signaling response take place, the network response is modified. This shows that processes similar to those occurring in classical LTD, notably characterized by induction and long-term maintenance of synaptic changes, can be studied in our *in vitro* system. Furthermore, the presence in the network of a sort of “memory” of exposure to NSs prompts us to speculate that the effects of these compounds may be also long-lasting *in vivo* and could have a physiological significance, since it renders the network insensitive to subsequent administration of the drug.

Tetrahydrodeoxycorticosterone and ALLO are potent allosteric modulators of GABAergic neurotransmission (Cooper et al., 1999), but at high concentrations, they also work as GABA_A_R agonists (Puia et al., 1990; Shu et al., 2004). In agreement with these properties, our analysis of the global excitability of the network showed a sharp increase in THDOC (**Figure 2**) and ALLO (**Figure 6**) inhibitory effects at concentrations between 100 and 1000 nM, probably due to a direct agonistic activity at GABA_A_Rs.

When the effect of ALLO was described using the “state analysis” (**Figure 6**), two apparently contrasting results were obtained: (i) in state 1, the excitability of both clusters of neurons decreased following a biphasic curve with a plateau between 10 and 300 nM and (ii) in state 2, the excitability of excitatory neurons increased at 2 nM by ~40% (**Figure 6B**). We believe the first effect could be ascribed to the presence of GABA_A_Rs with different affinities for ALLO or to the direct effect of the NS. The second result is in agreement with previous studies (Xiang et al., 1998) reporting that GABAergic drugs selective for the interneuronal GABA_A_Rs enhance excitability of intracortical circuits through disinhibition.

Many single-cell studies of the effects of ALLO and THDOC have been performed, but no differences were reported between their effects. We show here for the first time that THDOC and ALLO, although consistently producing network inhibition at high concentrations, when applied at low concentrations (10–100 nM) have different effects on excitatory and inhibitory clusters (**Figures 1A and 3** vs **Figure 6**). This finding suggests that ALLO may bind to different GABA_A_R isoforms with different affinity or that it activates some intracellular pathways only in specific cells. Furthermore, the network activity more easily recovered from ALLO than from THDOC, suggesting that the modulation produced by these drugs could be also different *in vivo*.

Our first network analysis of the NS effects provided information about the average changes in excitability of the network, but the states analysis highlighted important changes in the network connectivity: the appearance of heterogeneity in the activity during drug application. Several possible factors could account for the development of this observed heterogeneity. First, compounds that enhance GABAergic currents produced a uniform decrease in neuronal excitability in the whole network. This implies that all GABAergic synapses are similar in terms of receptor composition and/or function, which is known to be untrue. A second possibility is that a generalized tonic inhibition is exerted by low [GABA]_o_ acting on extrasynaptic receptors and producing, by membrane “shunting,” a hyperpolarized resting potential. This effect strongly reduces the probability that the membrane potential reaches the firing threshold and consequently increases the IBI. We could easily disregard this possibility because the low drug concentrations we used were unable to significantly alter IBI, and only high concentrations consistently silenced the networks. Furthermore, the balanced excitatory and inhibitory activity could produce either an increase or a decrease in global activity depending on the equilibrium among thousands of synaptic boutons, randomly active on pyramidal and interneuron cells. This factor is more likely to be responsible for the increase in heterogeneity after drug application, because a multitude of different isoforms of ligand-gated ion channels and, more importantly, different receptor densities, are present on the membranes of different neurons (Belelli and Lambert, 2005). We think that the rich repertoire of targets (and responses) generates an ample catalog of functional modes of connectivity leading to the heterogeneity of different states that we observed.

### PHASIC AND TONIC GABA INHIBITION

Previous studies suggested that tonic GABAergic currents are highly sensitive to NSs (Stell et al., 2003). Our experiments with GBZ were aimed at investigating this issue at the network level. GBZ, at concentrations that would be expected to only block the phasic GABAergic current, increased the activity of inhibitory neurons, leaving almost unaffected the excitatory neuron excitability. This suggests that interneurons are controlling each other mainly through a phasic inhibition. Several subclasses of GABAergic interneurons have been found (Ellender and Paulsen, 2010) with different inhibitory roles (Bacci and Huguenard, 2006), and differences in GABAergic neurotransmission onto glutamatergic cells and other GABAergic neurons have been reported. This fact explains why an increased activity of inhibitory neurons is not resulting in a decreased excitability of the excitatory neurons. By blocking the phasic GABAergic current with 100 nM GBZ, we expected to increase the network sensitivity to THDOC. In this condition, even though the NS still decreased network excitability, the sensitivity of the network to its effect was decreased by approximately one order of magnitude compared to the controls. The apparent inconsistency with the results previously obtained in hippocampal dentate gyrus and cerebellum granule cells in acute brain slices (Stell et al., 2003) probably depends on the fact that the GABA_A_R subunit composition responsible for tonic currents in their preparations was different, leading to different sensitivity to NSs. On the other hand, our data in cultured networks show an increased sensitivity to THDOC and BDZs as compared to similar studies done on adult thalamic slices (Cope et al., 2005).

### EFFECTS OF BENZODIAZEPINES ON NETWORK ACTIVITY

Benzodiazepines are widely used anxyolitic, hypnotic, sedative and anticonvulsant drugs, whose actions are mostly mediated by a potentiation of GABAergic neurotransmission. BDZs generally bind with high affinity to a specific site on the GABA_A_R, called the BDZ site, distinct from the NS modulatory site (Puia et al., 1992), as is the case for CLZ. Other BDZs, like MDZ, bind to the BDZ site and also act as agonists of the translocator protein (TSPO), to enhance the synthesis of steroids, including those which stimulate GABA_A_Rs (Papadopoulos et al., 2006).

In our experiments, CLZ and MDZ reduced network excitability with different potency (see **Table 1**). Interestingly, the effect of CLZ was almost completely reversible, whereas that of MDZ on inhibitory neurons was persistent, similarly to what happens after NS application. Tokuda et al. (2010) showed that MDZ inhibits LTD in rat hippocampal brain slices and that the effect was abolished by pre-treatment with FIN, an inhibitor of 5α-reductase, a key enzyme in NS synthesis. These authors concluded that endogenous NSs were implicated in the MDZ effect. To test this hypothesis, we pre-treated the cultures with FIN, but the long-lasting effect of MDZ on inhibitory neurons was not abolished (**Figure 9**). The discrepancy between our results and those of Tokuda et al. (2010), could derive from the different experimental models, i.e., cultures vs slices. It is also possible that different mechanisms underlie the appearance of LTD after treatment with MDZ in these preparations. Unexpectedly, CLZ, when applied after FIN treatment, produced a LTD of both excitatory and inhibitory clusters (**Figure 9**). A recent study showed that the anticonvulsant action of CLZ was reduced by FIN, indicating a possible contribution of NSs to the BDZ action (Dhir and Rogawski, 2011). Such interplay between NSs and CLZ in modulating network activity is also suggested by our results, although in our experiments, blocking the NS synthesis potentiated the BDZ effects.

**Table 1:** Effects of NSs (THDOC, ALLO) and BDZs (CLZ, MDZ) on excitability (EXC), engaged neurons (EngN), and long-term depression (LTD).

Drugs	THDOC	ALLO	CLZ	MDZ	FIN + CLZ	FIN + MDZ
**Reference figure**	**4**	**6B–D**	**8A–D**	**8E–H**	**9B**	**9B**
**[Drug] to silence network activity**	**>1 µM**	**>3 µM**	**>30 nM**	**>100 nM**		
**EXC state 1, [ ]**	↓	↓ **Biphasic**	↓↓	↓↓	↓↓	↓↓
**EXC state 1, LTD**	No	**+**−	No	Yes ++	Yes ++	Yes ++
**EXC state 1, [ ]**	↓↓	↓ **Biphasic**	↓↓	↓↓	↓↓	↓↓
**EXC state 1, LTD**	Yes +++	**+**−	No	Yes +++	Yes +++	Yes +++
**EXC state 2, [ ]**	↑	↑ to-plateau	↑ ↑	↑ − ↓	↑↑	↑ − ↓
**EXC state 2, LTD**	No	+	No	Yes +	Yes +	Yes +
**EXC state 2, [ ]**	↑	↑ to-plateau	↑	↑ − ↓	↑	↑ − ↓
**EXC state 2, LTD**	Yes ++	+	No	Yes +++	Yes +++	Yes +++
**ENGN state 1, [ ]**	–	–	–	–	–	–
**ENGN state 1, LTD**	No	No	No	No	No	No
**EngN state 1, [ ]**	–	–	–	–	–	–
**EngN state 1, LTD**	No	No	No	No	No	No
**EngN state 2, [ ]**	–	↓ **Biphasic**	↓	–	–	–
**EngN state 2, LTD**	No	No	No	No	No	No
**EngN state 2, [ ]**	–	↓ **Biphasic**	↓	–	–	–
**EngN state 2, LTD**	No	No	No	No	No	No

*EXC, EngN, and LTD of states 1 and 2 for excitatory (red) and inhibitory (black) neuron clusters are reported. In the last two columns CLZ and MDZ were applied after preconditioning with FIN. Arrows indicate the type of effect (↓, decrease; ↑, increase; –, no change) and the number of arrows indicate the level of the effect for each drug; the number of +’s indicate the duration of the LTD. Values in the second row indicate qualitatively, the average drug concentration that normally produced network silencing.*

On the whole, our data, summarized in **Table 1**, reveal the complexity of the network response to different GABA_A_R modulatory drugs at different concentrations. We believe that not only the excitability but also the number of recruited neurons are key factors in determining the effect of these drugs. Future studies will aim to look deeper into specific changes in the feedback and feed-forward connectivity rules in our system.

### DIFFERENT CONNECTIVITY MODES IN THE PRESENCE OF NEUROMODULATORS

The most common connectivity paradigms present in the central nervous system have been studied and discussed by many authors (recently reviewed by Grillner et al., 2005; Oren and Kullman, 2012). They have been specifically studied by using either classical or optogenetic procedures (Thomson et al., 2002; Lovett-Barron et al., 2012). Overall, it appears that various types of interneuronal feedback onto pyramidal neurons are functioning in immature and adult neocortex. It has been suggested that in the former case (very similar to our cultured networks), most of the simultaneously tested interneurons display a high probability of reciprocal connectivity, through chemical and electrical synapses as well as interneuron autapses (Gallareta and Hestrin, 1999; Gibson et al., 1999; Thomson et al., 2002; Bacci and Huguenard, 2006). Specific firing patterns recorded in selected neuronal populations encode information during physiological or pathological conditions and it is conceivable that changes in the connectivity induced by endogenously released compounds may modify the response of the system, when needed.

In conclusion, we believe that our analysis procedure better characterizes the number of functional states of a network and opens up the possibility of predicting the elementary “vocabulary” used by small networks of neurons (Luczak et al., 2009).

## Conflict of Interest Statement

The authors declare that the research was conducted in the absence of any commercial or financial relationships that could be construed as a potential conflict of interest.
